# Supplementary material for: Exploring the neurogenic differentiation of human dental pulp stem cells
Source: PLoS One. 2022 Nov 4;17(11):e0277134. doi: 10.1371/journal.pone.0277134 (PMC9635714; doi:10.1371/journal.pone.0277134)
Supplement: S1 Table — (PDF) [file pone.0277134.s001.pdf]

S1 Table: hDPSC information and stem cell characterization.

Reported Information	DPSCs (Lonza, Slough, UK)	DPSCs (Axol, Cambridge, UK)
**Product code**	PT-5025	ax3901
**Donor gender**	Male	Female
**Donor age**	16 yrs.	14 yrs.
**Stem cell markers**	≥ 90% positive for CD105, CD166, CD29, CD90 and CD73 markers.<br>≤ 10% positive for CD34, CD45, and CD133 markers.	> 90% positive for CD29, CD44, CD90, and CD105 markers.<br><10% positive for CD34 and CD45 markers.
**Multilineage differentiation validation**	–	Osteogenic, adipogenic, and chondrogenic differentiation.
**Virus and Microbiological testing**	Negative	Negative
**The culturing and sub-culturing materials to prepare a good cryovial stock.**	- DPSC BulletKit™ Medium (PT-3005, Lonza) supplied as 1 ml/5 cm^2 includes:<br>DPSC basal Medium (PT-3927, Lonza),<br>DPSC Growth Medium (PT-4516): contains 50 ml DPSC growth supplement, 10 ml L-glutamine, 5.0 ml Ascorbic Acid, and 0.5 ml Gentamicin/Amphotericin-B<br>-Reagent Pack™ Subculture Reagents (CC-5034, UK): HEPES Buffered Saline Solution, 0.25% Trypsin/EDTA, and Trypsin Neutralizing Solution (TNS).	- Alpha-modified minimum essential medium (α-MEM) (Biosera, UK) with 2 mM L-glutamine, 1% antibiotics (penicillin/streptomycin (100 IU.ml^{-1}), and 10% FBS.<br>- Standard sub-culturing materials; (0.25% Trypsin/EDTA, and 10% FBS complete media).
