# Supplementary material for: Exploring the neurogenic differentiation of human dental pulp stem cells
Source: PLoS One. 2022 Nov 4;17(11):e0277134. doi: 10.1371/journal.pone.0277134 (PMC9635714; doi:10.1371/journal.pone.0277134)
Supplement: S2 Table — (PDF) [file pone.0277134.s002.pdf]

S3 Table: The antibodies and dilutions used for immunocytochemical analysis

Antibody	Type	Host species	Description	Dilution	Reactivity	Product number	Supplier
**Anti-βIII-tubulin (2G10)**	Primary	Mouse	Monoclonal	1:500	Human, mouse, rat, rabbit, chicken, cow, cat, quail	ab78078	Abcam, UK
**Anti-160 kD Neurofilament Medium**	Primary	Rabbit	Polyclonal	1:1000	Human, rat, cow	ab9034	Abcam, UK
**Antti-GFAP**	Primary	Rabbit	Monoclonal	1:500	Human, rat	ab33922	Abcam, UK
**Alexa Fluor 488**	Secondary	Goat	Monoclonal	1:400	Anti-mouse	A20181	Invitrogen, Thermo Fisher Scientific, UK
**Alexa Fluor 555**	Secondary	Goat	Polyclonal	1:500	Anti-Rabbit	ab150078	Abcam, UK
