# Supplementary material for: Exploring the neurogenic differentiation of human dental pulp stem cells
Source: PLoS One. 2022 Nov 4;17(11):e0277134. doi: 10.1371/journal.pone.0277134 (PMC9635714; doi:10.1371/journal.pone.0277134)
Supplement: S3 Table — (PDF) [file pone.0277134.s003.pdf]

**S4 Table: The primer details used for real-time PCR.**

| Gene | Primer sequence (5→3) | Amplicon size (bp) | Accession number (NCBI) | Efficiency value | |
|---|---|---|---|---|---|
| | | | | SH-SY5Y | DPSCs |
| **Housekeeping genes** | | | | | |
| GAPDH | (F) CTCCTGTTCGACAGTCAG<br>(R) GCCCAATACGACCAAATC | 111 | NM_002046.7 | 1.70 | 1.80 |
| RPL13A | (F) CCTGGAGGAGAAGAGGAAAGAGA<br>(R) TTGAGGACCTCTGTGTATTTGTCAA | 126 | NM_012423.4 | 1.90 | 1.70 |
| B2M | (F) ACCCCCACTGAAAAAGATGA<br>(R) ATCTTCAAACCTCCATGATG | 114 | NM_004048.3 | 1.60 | 1.90 |
| HPRT1 | (F) GACCAGTCAACAGGGGACAT<br>(R) AACACTTCGTGGGGTCCTTTTC | 195 | NM_000194.3 | 1.90 | 1.70 |
| **Target genes** | | | | | |
| ENO2 (NSE) | (F) CTGAAGCCATCCAAGCGTGC<br>(R) CCCACCACCAGGTCAGCAAT | 109 | NM_001975.2 | 1.85 | 1.92 |
| NES | (F) GAGAACTCCCGGCTGCAAAC<br>(R) CCAGCTTGGGGTCCTGAAAG | 70 | NM_006617.1 | 1.83 | 1.93 |
| SYN1 | (F) AATACTGGCTCTGCGATGCT<br>(R) TGACCACGAGCTCTACGATG | 223 | NM_006950.3 | 1.94 | 1.78 |
| GFAP | (F) CCATTCCCGTGCAGACCTTC<br>(R) TCTGAGAGGCAGGCAGCTAAC | 180 | NM_0011310 19.2 | 2.07 | 1.60 |
| PRPH | (F) AGATCGCCACCTACCGCAAG<br>(R) CCCATTCCGGGTCTCAATGGT | 170 | NM_006262.3 | 2.08 | 1.85 |
| SCN9A | (F) GGCAACTTCTGATGACAGCG<br>(R) GTGCAAATCTGTACCACCAAGG | 200 | NM_0013655 36.1 | 2.01 | 2.05 |
| DBH | (F) CAGATATCTCCGCCTGGAAG<br>(R) TGCAGTAGCCAGTGAGGATG | 197 | NM_000787.3 | 1.76 | _ |
| POU4F1 | (F) TTGCCATGCATCCCACCCTC<br>(R) TGAAAGGATGGCTCTTGCCCT | 198 | NM_006237.3 | 2.03 | 1.60 |
| CHAT | (F) TCCAACGAGGACGAGCGTTT<br>(R) ATCATGTCCAGCGAGTCCCG | 122 | NM_0011429 34.1 | 2.10 | 1.93 |

| | | | | | |
|---|---|---|---|---|---|
| ACHE | (F) CCTCCTTGGACGTGTACGAT<br>(R) AAACAGCGTCACTGATGTCG | 218 | NM_001302622.2 | 1.64 | 1.72 |
| GAP43 | (F) CAGAATTAAAAGGGAACCTGG<br>(R) TGTGACTCATTTTCCTTGTG | 93 | NM_001130064 | 2.04 | 2.10 |
| MNX1 | (F) GTTCAAGCTCAACAAGTACC<br>(R) GGTTCTGGAACCAAATCTTC | 98 | NM_001165255 | 1.65 | 1.88 |

Abbreviations: GAPDH (glyceraldehyde-3-phosphate dehydrogenase); RPL13A (ribosomal protein L13A); B2M (beta-2-microglobulin); HPRT1 (hypoxanthine phosphoribosyltransferase 1); ENO2/NSE (enolase 2/neuron-specific enolase); NES (nestin); SYN1 (synapsin I); GFAP (glial fibrillary acidic protein); PRPH (peripherin); SCN9A (voltage-gated sodium channel alpha subunit 9); DBH (dopamine beta-hydroxylase); POU4F1 (POU class 4 homeobox 1); CHAT (choline O-acetyltransferase); ACHE (acetylcholinesterase); GAP43 (growth associated protein 43); MNX1 (motor neuron and pancreas homeobox 1).
