# Supplementary material for: Exploring the neurogenic differentiation of human dental pulp stem cells
Source: PLoS One. 2022 Nov 4;17(11):e0277134. doi: 10.1371/journal.pone.0277134 (PMC9635714; doi:10.1371/journal.pone.0277134)
Supplement: S1 File — (PDF) [file pone.0277134.s004.pdf]

Neurogenic differentiation protocol for neuroblastoma cell line (SH-SY5Y) and human dental pulp stem cells (hDPSCs)

1 Preparing the cell cultures for neuronal differentiation:

1.1 SH-SY5Y and hDPSCs were cultured in T75 flask with complete growth media (see **Table 1**) and then the cells were incubated in a humidified incubator at 37 °C and 5% CO_2 (Heracell 150i, Thermo Scientific, UK). Media change was performed every 3-4 days till reaching ~ 80 % cell confluence.

Table 1: The composition of the complete growth media

	SH-SY5Y	hDPSCs
**Complete growth media**	- 10 ml Dulbecco's modified Eagle's medium/Ham's nutrient mixture F12 (DMEM/F12), Sigma Aldrich, UK.<br><br>- 1% antibiotics: penicillin/streptomycin (100 $IU.ml^{-1}$), Sigma-Aldrich, UK.<br><br>- 10% fetal bovine serum (FBS), Biosera, UK.	- Alpha-modified minimum essential medium (α-MEM), Biosera, UK.<br><br>- 1% antibiotics: penicillin/streptomycin (100 $IU.ml^{-1}$), Sigma-Aldrich, UK.<br><br>- 2 mM L-glutamine, Sigma-Aldrich, UK.<br><br>- 10% foetal bovine serum (FBS), Biosera, UK.

Note: hDPSCs should be cultured and incubated separately in a specific incubator for primary cells to avoid any abnormal changes or contamination from the cell line. It is preferred to firstly do the work for the hDPSCs and incubate them and then do the similar work for SH-SY5Y cells.

1.2 The cells were detached with 0.25% trypsin-ethylenediaminetetraacetic acid (Trypsin-EDTA) treatment, then neutralized and centrifuged as described for each cell type in **Table 2**.

Table 2: Trypsin-EDTA treatment, neutralizing and centrifugation.

Step	SH-SY5Y	hDPSCs
Trypsinization amount, and time, and condition	3-5ml incubated for 1-2 minutes at incubator.	3-5ml incubated for 3 minutes at incubator.
Neutralizing media	6-10ml (double amount) of its complete growth media mentioned in **Table 1**.	6-10ml (double amount) of its complete growth media mentioned in **Table 1**.
Centrifuge speed and time	1100 rpm (220 x g) for 3 min	1100 rpm (220 x g) for 4 min

Notes:

- The SH-SY5Y cells have 2 different cell types (N-type detached and adherent S-type cells). Hence, the supernatant media was transferred to 50 ml falcon tube as it may contain the N-type detached cells "the more neurogenic potential cells", and then the remaining adherent S-type cells were detached with Trypsin-EDTA treatment as described above in **Table 2**.
- More cells were detached with gentle tapping on the bottom of T75 flask in the last minute of trypsinization incubation time. In this protocol, the time of trypsinization is short to avoid the harsh effect of trypsin/EDTA on the cells if incubate for longer time and detaching more cells is performed by gentle tapping on the bottom of the flask.

1.3 After centrifuge step, the supernatant was aspirated and the cells were suspended with 2 ml of complete growth media. Cell counting was then conducted.

1.4 The desirable cell density was seeded (5000 cells/cm^2 for SH-SY5Y, and 625 cells/cm^2 for hDPSCs) per well of the collagen coated six-well plates (Thermo Fisher Scientific, UK), laminin/collagen-coated coverslips (Electron Microscopy Sciences, UK) or T25 flasks (Thermo Fisher Scientific, UK) using the complete growth media (~ 3ml per well and 5-6ml per T25 flask).

Note: The selection of the seeding surface (coated or non-coated) and surface area (6-well plate, T25 flask or coated coverslips) is depending on the assay. For example, T25 flask without coating is chosen for PCR assay to get more and pure RNA as it has more surface area bigger than that of well of 6-well plate and no contaminations from the coating materials.

1.5 The cells were incubated at temperature of 37 °C, and 5% CO_2 concentration “humidified incubator” for overnight to allow cell attachment to collagen/laminin coat on the floor of the well.

2 Preparing the differentiating supplements:

2.1 The stock solutions of differentiating supplements were prepared which are all trans-retinoic acid (ATRA) and brain derived neurotrophic factor (BDNF) as follows:

A. ATRA supplement (R2625, Sigma-Aldrich, UK)

The ATRA product (powder) was dissolved in sterilized pure dimethyl sulfoxide (DMSO; D2650, Sigma-Aldrich, UK) to have a stock solution with specific molarity of 10 mM. According to manufacture instructions, to prepare 0.01M “10 mM” stock solution, you need 3 mg/ml. For example, the ordered product was 50 mg, so it should be dissolved in 16.67 ml of the solution “DMSO”.

Hence, the steps to prepare 10 mM stock of ATRA are as follows:

I. 16.67 ml (16 ml plus 670 µl) of sterilized DMSO was used to dissolve the 50-mg powder of ATRA.
II. The mixture was vortexed to get a homogenous mixing for 10-15 seconds using vibrating shaker device.
III. The mixture “the prepared ATRA solution” was filtered using Nylon Microfilter 0.2 µm to ensure that the solution clean “no contamination has occurred during the mixing procedure”.
IV. 0.5-ml aliquots were stored in dark or black Eppendorf tubes (0.5 ml/Eppendorf), or you can use any light protected vials and then kept at -20 °C till use.
V. At the time of differentiation experiment, the ATRA solution was diluted with culture medium right before use “with well and gentle pipetting”.

Notes:

- ATRA is highly sensitive to light, air in solution, and heat. In addition, solutions of ATRA in pure organic solvents are reasonably stable when stored in the dark whereas in aqueous solutions are degraded quickly. Consequently, the following precautions should be taken in consideration:
- All solution preparations should be performed in subdued light of the laboratory room and cabinet hood's lights are switched off.
- ATRA was stored in sterilized dark or black Eppendorf tubes or can be stored in any light protected vials at -20 °C as previously mentioned.
- The plates/flasks were covered with sterilized tin foil to protect the cell cultures from being exposed to the light inside incubator during differentiation time with ATRA supplement. The tin foil was sterilized with UV light in the hood before starting the experiment.
- To avoid air bubbles formation that may affect the ATRA efficiency, the ATRA was mixed with culture media using gentle pipetting and then also gentle supplying the media to cell culture.
- Any heat source should be avoided that may damage the ATRA chemical structure.
- The ATRA should be immediately defrosted and freshly prepared with the culture medium right before the use in every media change.

B. BDNF supplement (78005, STEMCELL TECHNOLOGIES; SRP3014, Sigma-Aldrich, UK)

The BDNF material was reconstituted according to manufacture instructions as following steps:

I. Centrifuge vial before opening to collect the BDNF powder at the bottom of the vial.

II. Reconstitute the BDNF powder “10 µg” in sterile water to make up a concentration of at least 0.1 mg/ml by gentle pipetting the solution down the walls of the vial. For example, to get concentration of 0.1 mg/ml, you need to reconstitute 10 µg powder in 100 µl of sterile water (Molecular biology grade water, 46-000-CV, Corning, USA).

III. Aliquot the reconstituted solution into small sterile vials (20µl) and kept in -20 °C till use.

Notes:

- Do not vortex the solution upon the BDNF reconstitution.
- All the above steps should be performed as fast as possible to avoid degradation of the BDNF material.
- Avoid multiple freezing and thawing as the BDNF material may be degraded due to repeated freezing and thawing. Always defrost the aliquoted vial (20µl) for single use and directly use it for the experiment.
- Do not keep the reconstituted solution in frost-free freezer.

3 Neurogenic differentiation procedures

All details of the experimental groups regarding medium type, supplementations, and duration of the treatments are described in **Table 3** whereas the timeline of media change is shown in **Figure 1**.

Table 3: Experimental groups (differentiated and control groups)

<table>
<tr><th>Experimental groups</th><th>Culturing medium and supplementations</th><th>Incubation time (days)</th><th>Total (days)</th></tr>
<tr><td>Control (standard cell culture)</td><td>10% FBS DMEM/F12* supplemented with 10 µM DMSO$.</td><td>12</td><td>12</td></tr>
<tr><td>ATRA</td><td>10% FBS DMEM/F12* supplemented with 10 µM ATRA.</td><td>12</td><td>12</td></tr>
<tr><td rowspan="2">ATRA→BDNF</td><td>1st stage: 10% FBS DMEM/F12* supplemented with 10 µM ATRA.</td><td>5</td><td rowspan="2">12</td></tr>
<tr><td>2nd stage: Serum-free DMEM/F12* supplemented with 50 ng/ml BDNF.</td><td>7</td></tr>
<tr><td rowspan="2">ATRA→ 0 serum (2nd control)</td><td>1st stage: 10% FBS DMEM/F12* supplemented with 10 µM ATRA.</td><td>5</td><td rowspan="2">12</td></tr>
<tr><td>2nd stage: Serum-free DMEM/F12* without any supplements.</td><td>7</td></tr>
</table>

*Supplemented with penicillin/streptomycin
$DMSO is added as it is the dissolvent used to prepare the ATRA, so the control group is identical to differentiating group but without the differentiating supplement.

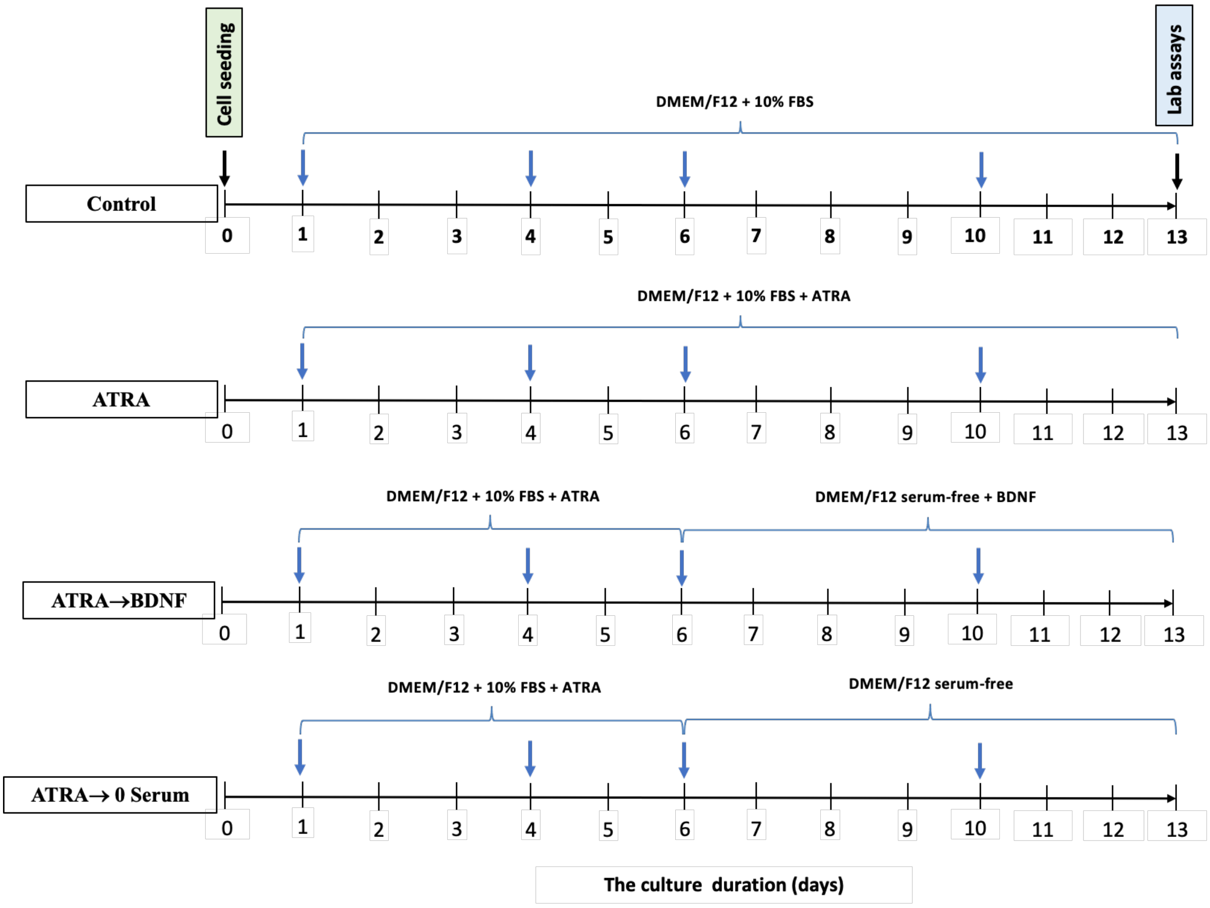

Figure 1: The timeline and frequent media change for the experimental groups. Blue arrows denote time of the change media whereas the black arrows denote time of starting and ending of the experiment.

The procedures for the neurogenic differentiation are:

3.1 The differentiation and control media were freshly prepared by diluting the stock solution with culture medium to get the desirable concentration as follows:

- For final concentration of 10 µM ATRA in culture media (0.01 % DMSO v/v), take 1µl/1ml (1 µl of 10 mM ATRA stock is added to 1 ml of culture media) which means 15 µl of 10 mM stock used for each 15 ml culture media (10µM)).
- For 50 ng/ml concentration of BDNF in the culture media from 0.1 mg/ml stock solution, you need 1µl of BDNF for every 2 ml of culture medium. For example, 7.5 µl of BDNF solution are diluted in 15 ml of culture medium.

Notes:

- This can be easily calculated using the dilution calculator of the available free molarity calculators. There are multiple free molarity calculators in the websites; we used this calculator https://www.graphpad.com/quickcalcs/molarityform/.
- All supplements were defrosted and immediately used for the experiment to avoid the material degradation over time because of being in aqueous solution.

3.2 The overnight media was aspirated form each well/flask, and then the prepared differentiating (ATRA-supplemented media) or control media as described in **Table 3** were directly supplied into the culturing well/flask. Then, the cell cultures were incubated at a humidified incubator at 37 °C and 5% CO_2 (Heracell 150i, Thermo Scientific, UK) till the next media change (2-3 days later).

Notes:

- As previously mentioned, the ATRA supplement is sensitive to the light, so this media change was performed in hood's light off and room's subdued light.
- To protect the cell cultures from being exposed to the light which can damage the ATRA structure and subsequently affecting the differentiation, the plates/flasks were covered with sterilized tin foil.

3.3 Subsequent media changes were performed after 2-3 days with ATRA-supplemented or control media and the plates/flasks were incubated at the humidified incubator at 37 °C and 5% CO_2.

3.4 After 5 days with and without ATRA supplementation, the cell cultures were washed twice with blank media without any supplementation to remove the remaining FBS and ATRA before starting the 2nd step of differentiating protocol for ATRA→BDNF and ATRA→ 0 serum groups which is either supplementing with and without 50 ng/ml BDNF in the serum-free media, respectively.

3.5 The BDNF-supplemented serum-free media was supplied into the cell culture wells of the planned ATRA→BDNF group and the serum-free media alone was supplied into the cell culture wells of its parallel control group (ATRA→ 0 serum). The other two experimental groups (control and ATRA) were supplied with and without ATRA-supplemented media as previously described in **Table 3**. Then, all cell culture groups were incubated at the humidified incubator till the next change after 3-4 days.

3.6 After 3-4 day of incubation, the media per experimental group was changed as previously mentioned in step 3.5 and then the experimental groups were incubated at the humidified incubator for additional 3-4 days before the end of the 2nd stage of the neurogenic differentiation period (7 days).
